# Identification of immune cell infiltration and effective biomarkers of polycystic ovary syndrome by bioinformatics analysis

**DOI:** 10.1186/s12884-023-05693-4

**Published:** 2023-05-24

**Authors:** Mengge Gao, Xiaohua Liu, Mengxuan Du, Heng Gu, Hang Xu, Xingming Zhong

**Affiliations:** 1 NHC Key Laboratory of Male Reproduction and Genetics, Guangdong Provincial Reproductive Science Institute (Guangdong Provincial Fertility Hospital), Guangzhou, 510600 China; 2 Department of Public Health and Preventive Medicine, School of Medicine, Jinan University, Guangzhou, Guangdong Province China; 3 Department of Clinical Nutrition, Huadu District People’s Hospital, Southern Medical University, 48 Xinhua Road, Huadu, Guangzhou, 510800 Guangdong China

**Keywords:** Polycystic ovary syndrome, Immune cell infiltration, Biomarker, Single-sample gene set enrichment analysis

## Abstract

**Background:**

Patients with polycystic ovary syndrome (PCOS) exhibit a chronic inflammatory state, which is often accompanied by immune, endocrine, and metabolic disorders. Clarification of the pathogenesis of PCOS and exploration of specific biomarkers from the perspective of immunology by evaluating the local infiltration of immune cells in the follicular microenvironment may provide critical insights into disease pathogenesis.

**Methods:**

In this study, we evaluated immune cell subsets and gene expression in patients with PCOS using data from the Gene Expression Omnibus database and single-sample gene set enrichment analysis.

**Results:**

In total, 325 differentially expressed genes were identified, among which *TMEM54* and *PLCG2* (area under the curve = 0.922) were identified as PCOS biomarkers. Immune cell infiltration analysis showed that central memory CD4^+^ T cells, central memory CD8^+^ T cells, effector memory CD4^+^ T cells, γδ T cells, and type 17 T helper cells may affect the occurrence of PCOS. In addition, *PLCG2* was highly correlated with γδ T cells and central memory CD4^+^ T cells.

**Conclusions:**

Overall, *TMEM54* and *PLCG2* were identified as potential PCOS biomarkers by bioinformatics analysis. These findings established a basis for further exploration of the immunological mechanisms of PCOS and the identification of therapeutic targets.

**Supplementary Information:**

The online version contains supplementary material available at 10.1186/s12884-023-05693-4.

## Introduction

Polycystic ovary syndrome (PCOS) is a common reproductive endocrine metabolic disease in women of childbearing age and is often characterised by chronic anovulation and hyperandrogenaemia. Its clinical manifestations include menstrual disorders, hirsutism, acne, and polycystic ovarian changes, and these symptoms can be accompanied by metabolic diseases, such as obesity, insulin resistance (IR), and dyslipidaemia [1]. However, the pathogenic mechanisms of PCOS are still unclear. In addition to the ovarian-pituitary-hypothalamic-gonadal axis, studies of PCOS pathogenesis must also consider ovarian local cytokines, immunology, and genetics.

Recent studies have found that the inflammatory immune mechanism is closely related to the occurrence and development of PCOS. Under physiological conditions, appropriate inflammatory stress is conducive to the growth and development of oocytes [2, 3]. However, under pathological conditions, the inflammatory response is enhanced, the development of oocytes may be limited, and the further development of chronic inflammation leads to decreased ovum quality, thereby affecting ovulation [4]. Systemic and ovarian cytokines (e.g., tumour necrosis factor [TNF]-α, interleukin [IL]-6, and IL-18) can change the local microenvironment in the ovary, regulate ovarian function, induce excessive androgen production, and promote IR through various mechanisms [5]. Inflammation in the follicular microenvironment may be involved in the dysfunction of the hypothalamic-pituitary-gonad axis and the occurrence and development of follicular dysplasia.

In studies of the distribution of white blood cells in the ovary and cytokine mRNA expression in the follicular fluid (FF) of patients with PCOS and non-PCOS women undergoing fertilisation embryo transfer in vitro, Wu et al. [6] showed that T lymphocytes play important roles in the local pathological mechanisms of PCOS. T lymphocytes secrete various inflammatory and immunomodulatory molecules that participate in the regulation of ovarian function. T-cell subsets have also been shown to be dysregulated in the peripheral blood and ovaries of patients with PCOS owing to disruption of sex hormone levels in these patients [7].

The pathogenesis of PCOS is multifactorial and complex. In addition to reproductive abnormalities, the pathogenic mechanisms also include interactions between the immune system and reproduction, resulting in a variety of changes to cytokines and immune cells. Therefore, evaluation of immune cell infiltration in patients with PCOS based on changes in the expression levels of genes may be essential for elucidating the immunological mechanisms of PCOS and identification of novel biomarkers.

In this study, we aimed to identify the roles of immune cell subsets and related gene expression changes in the pathogenesis of PCOS. We downloaded four PCOS datasets from the Gene Expression Omnibus (GEO) database and analysed differentially expressed genes. Two different machine learning algorithms were then used to further identify PCOS biomarkers. We also studied PCOS from the perspective of immunology using single-sample gene set enrichment analysis (ssGSEA) to evaluate the differences in the compositions of 28 immune cell subsets between patients with PCOS and healthy women of reproductive age. In addition, the relationships between PCOS biomarkers and immune cell infiltration were studied to improve our understanding of the immunological mechanisms of PCOS occurrence and development.

## Materials and methods

### Data downloading

First, the “*GEOquery*” package in R software (version 3.6.1, http://r-project.org/) was used to download the PCOS expression profile datasets GSE84958, GSE106724, GSE137684, GSE114419 and GSE193812 from the GEO database (https://www.ncbi.nlm.nih.gov/geo/). A description of all databases is presented in Supplementary Table S1.

### Data preprocessing

We combined the GSE84958, GSE106724, GSE137684, and GSE114419 gene expression matrices, and interbatch differences were removed using the “*sva*” package [8]. Boxplots [9] and two-dimensional principal component analysis (PCA) cluster plots were used to visualise the effects of removing interbatch differences. The flow chart for this study is shown in Supplementary Figure S1.

### Evaluation of the distribution of immune cell subtypes

ssGSEA was used to quantify the infiltration of immune cells in patients with PCOS. The abundances of the following 28 types of immune cells were obtained from the R package “GSVA” [10]: activated B cells, activated CD4^+^ T cells, activated CD8^+^ T cells, activated dendritic cells (DCs), CD56^+^ natural killer (NK) cells, CD56^−^ NK cells, central memory CD4^+^ T cells, central memory CD8^+^ T cells, effector memory CD4^+^ T cells, effector memory CD8^+^ T cells, eosinophils, γδT cells, immature B cells, immature DCs, mast cells, myeloid inhibitory cells, memory B cells, monocytes, NK cells, NK T cells, neutrophils, plasmacytoid DCs, macrophages, regulatory T cells (Tregs), follicular helper T cells, type 1 T helper (Th1) cells, type 17 T helper (Th17) cells, and type 2 T helper (Th2) cells.

### Screening of differentially expressed genes (DEGs)

DEGs were filtered through the “*limma*” package [11]. The DEG threshold point was an adjusted *P* value less than 0.05 and a |log_2_ fold change| greater than 0.5.

### Functional enrichment analysis

The gene names of DEGs were converted to gene IDs using the R package “org.hs.eg.db”. Gene ontology (GO), Kyoto Encyclopaedia of Genes and Genomes [12, 13] (KEGG), and disease ontology (DO) analyses were performed using the R package “clusterProfiler” [14], and *p* value correction from multiple trials was performed using the BH method. GO, DO, and KEGG pathways with significant enrichment of DEGs were screened. GO annotations of DEGs included cellular components, biological processes, and molecular functions, which were used to analyse the functional enrichment of DEGs.

**Fig. 1:**
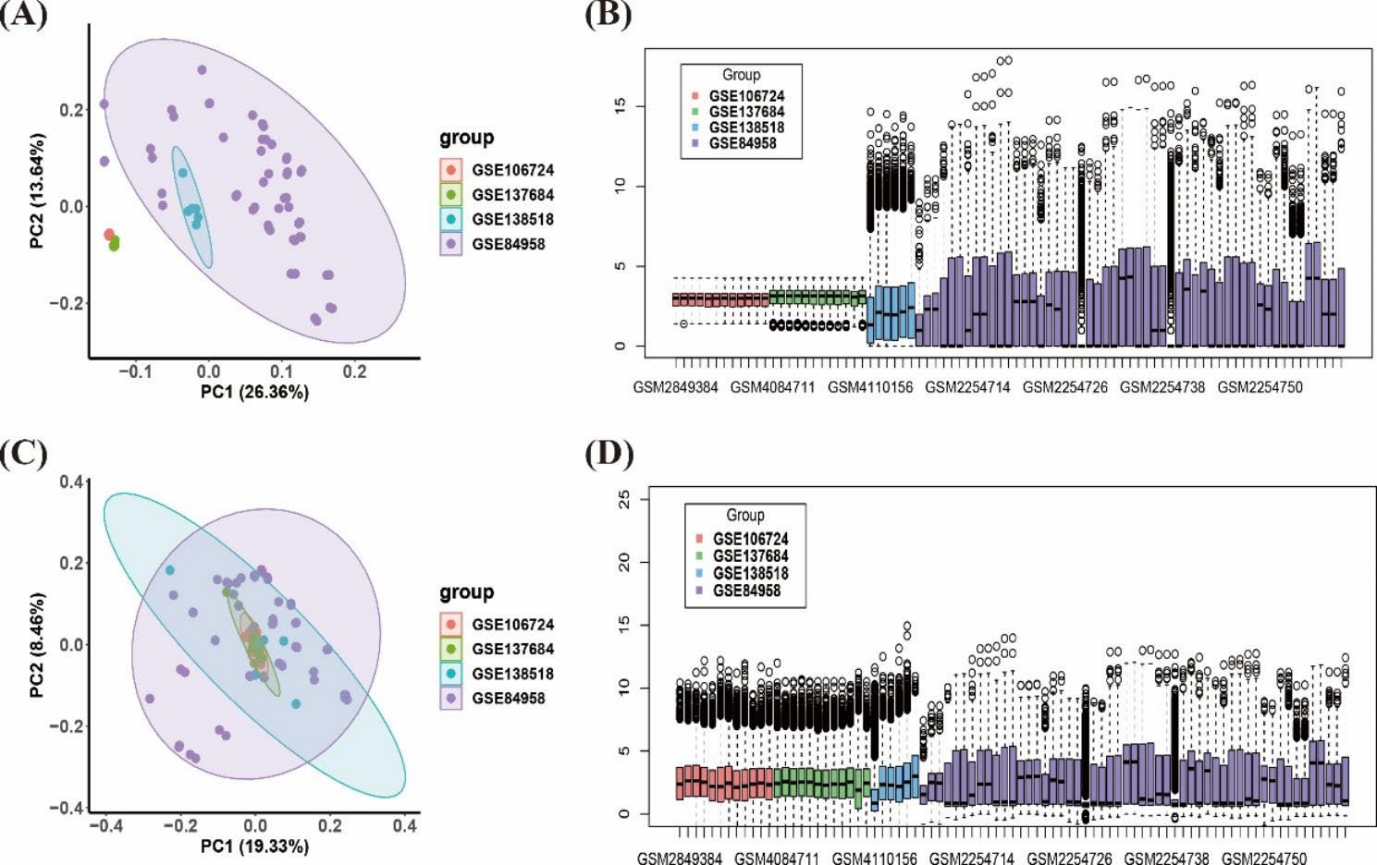
Two-dimensional PCA and boxplot before and after removing interbatch effects. (**A**, **B**) Data before removing interbatch effects in datasets GSE84958, GSE106724, GSE114419, and GSE137684. (**C**, **D**) Data after removing interbatch effects

### Screening of biomarkers

Protein-protein interaction (PPI) network results for DEGs were obtained using STRING, and LASSO regression and BORUTA algorithm analyses were used for feature selection to screen biomarkers of PCOS. The LASSO algorithm was implemented through the R package “glmnet” [15]. The BORUTA algorithm was implemented through the R package “Boruta” [16]. Similar to a random forest classifier, this approach reduced the error caused by random fluctuation and correlation by adding randomness to the system and collecting results from a random sample set. The candidate biomarkers obtained using the two methods were intersected using a Venn diagram to extract the final candidate biomarkers.

**Fig. 2:**
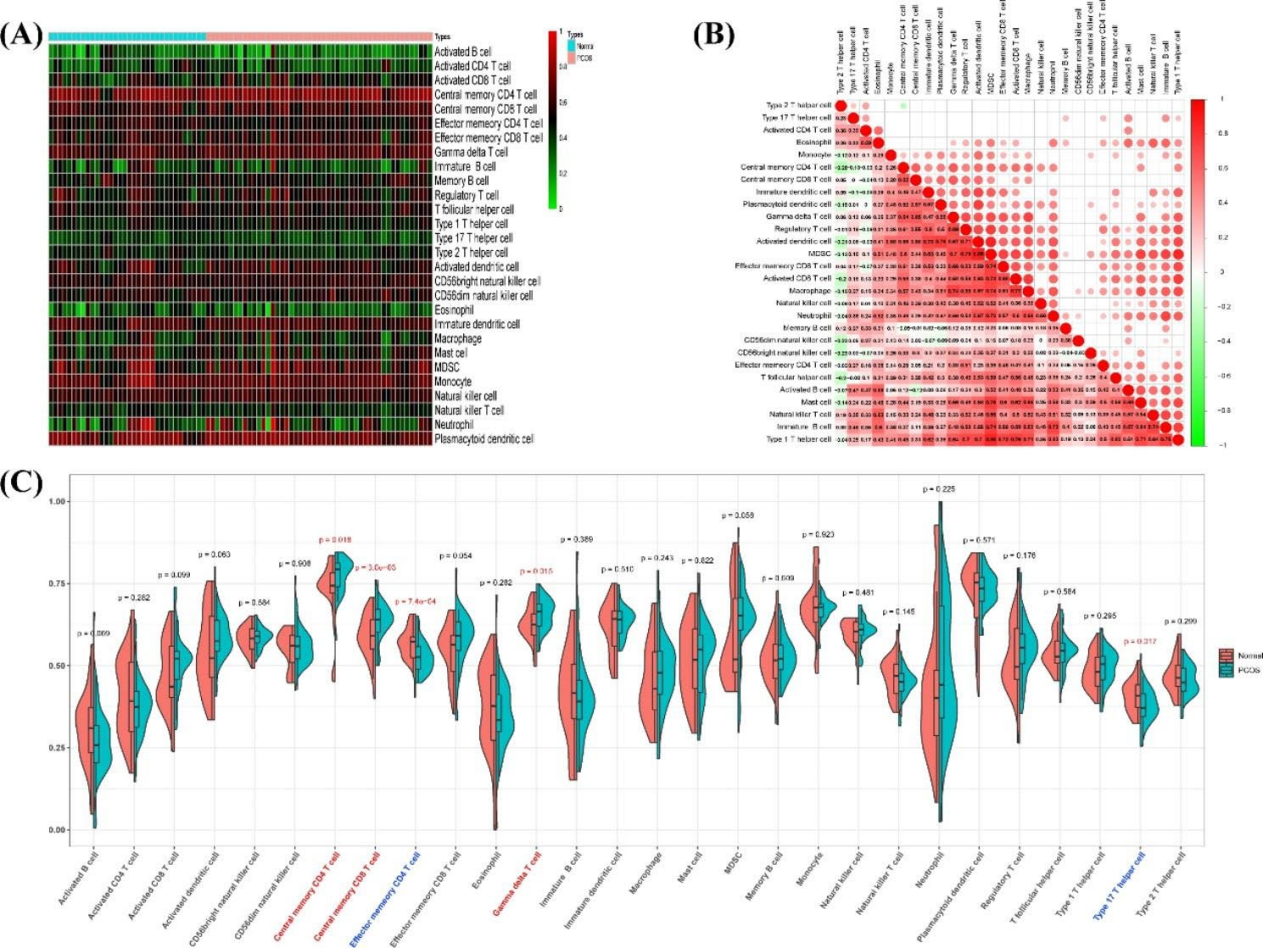
Differences in immune cell infiltration between patients with PCOS and normal controls. (**A**) Differences in the enrichment of immune cells between samples from the two groups. The normalised relative abundances of immune cells in each sample are shown, with red representing high enrichment degree and green representing low enrichment degree. (**B**) Correlations of 28 types of immune cells in the dataset. Colour blocks with a correlation coefficient *p* value greater than 0.05 in the upper part of the graph are not displayed; red represents a positive correlation, and green represents a negative correlation, with darker colour indicating a stronger correlation. (**C**) Differences in the enrichment of 28 types of immune cells between patients with PCOS and normal controls. Results with *p* values less than 0.05 are indicated in red, and immune cells with different enrichment degrees on the axis are indicated as red or blue. Red indicates a significant increase in the PCOS group, whereas blue indicates a significant decrease in the PCOS group

### Correlation analysis between biomarkers and immune cells

Spearman rank correlation analysis was used to investigate the correlations between the selected biomarkers and the level of immune cell infiltration.

### Analysis of the predictive value of biomarkers

In order to determine the predictive value of biomarkers for PCOS, receiver operating characteristic (ROC) curve analysis was carried out to explore the sensitivity and specificity of candidate biomarkers for PCOS prediction. Screening of the ROC area under the curve (AUC) values identified the dataset that could distinguish PCOS from normal control samples. An external data set and some peripheral blood samples were then used for testing. In this study, 6 PCOS patients who visited Guangdong Provincial Fertility Hospital from March 2022 to September 2022 were included and 6 women of reproductive age who visited the hospital and passed physical examination during the same period were included as the control group. Using peripheral blood RNA as template, cDNA was synthesized using Evo M-MLV reverse transcription reagent premix configuration system. Three groups of replicates were set for each sample, 2^-ΔΔCT^ formula was used to calculate the relative change of each gene expression in each sample.

### Statistical analysis and graphical visualisation

All analyses in this paper were carried out by R version 4.0.5. The analysis results were visualised using the R packages “corrplot” [17], “ggplot2” [18], and “pheatmap” [19] and spliced with “patchwork”.

## Results

### Data preprocessing

In this study, we selected 83 samples from four different datasets, including 34 PCOS samples and 49 normal control samples. A two-dimensional PCA diagram and boxplot showed the effects before and after treatment (Fig. 1A–D). The results demonstrated that clustering of the two groups of samples was more obvious after normalisation, indicating that differences between batches had been eliminated and that the samples were from reliable sources.

**Fig. 3:**
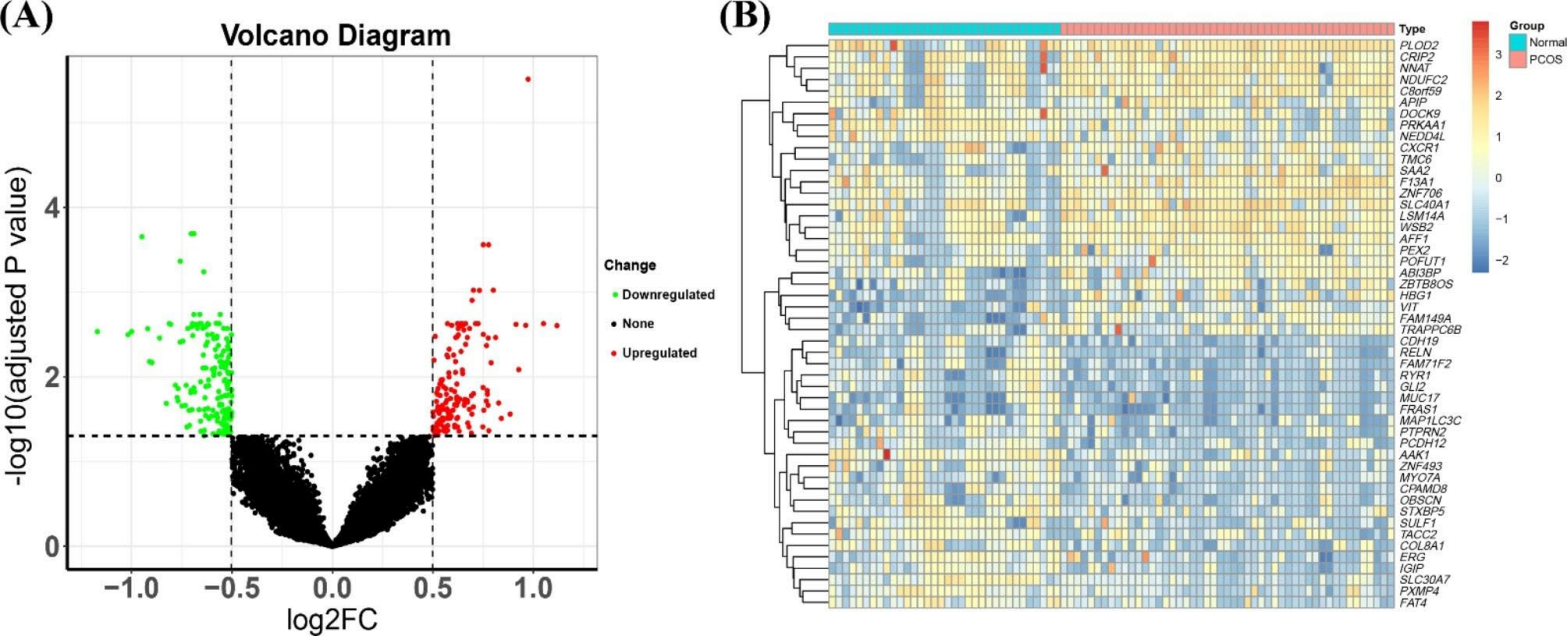
Analysis of DEGs. (**A**) Volcano diagram of DEGs between patients with PCOS and normal controls. Green represents significantly downregulated genes in the PCOS group, red represents significantly upregulated genes in the PCOS group, and black represents genes with no difference between groups. (**B**) Heat map of the top 50 genes with larger absolute value differences in expression between sample groups. The colour of the block represents the normalised expression of corresponding genes in the sample

**Fig. 4:**
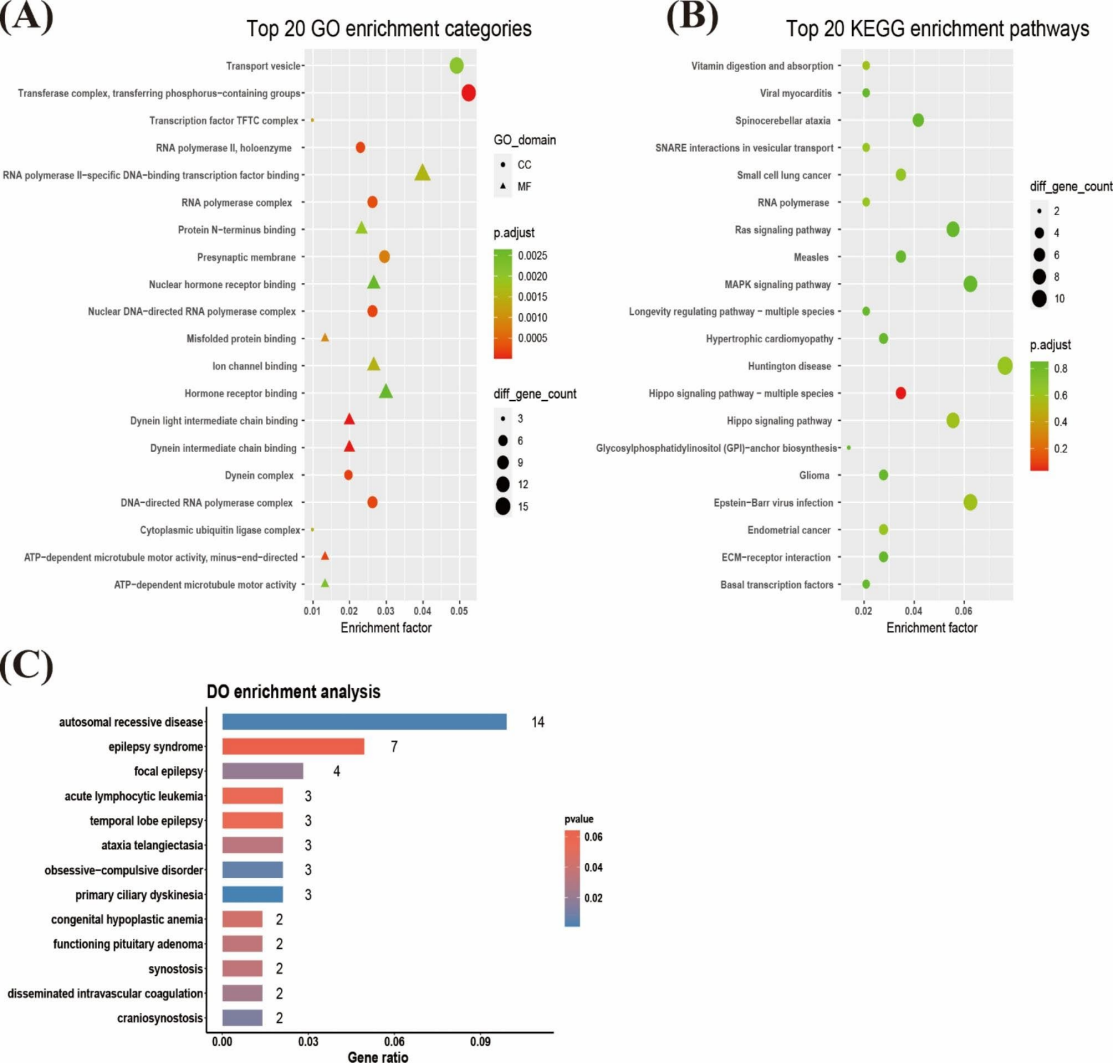
Functional correlation analysis of DEGs. (**A**) Top 20 results from GO enrichment analysis of DEGs between the PCOS and normal control groups. The shape of the pattern indicates the annotation of GO, the size of the pattern indicates the number of enriched genes, and the colour of the pattern indicates the *p* value. (**B**) Top 20 results from KEGG enrichment analysis of DEGs between the PCOS and normal control groups. The size of the pattern indicates the number of enriched genes, and the colour of the pattern indicates the *p* value. (**C**) Partial results from DO analysis of DEGs between the PCOS and normal control groups. The abscissa indicates the number of enriched genes, and the colour indicates the *p* value

**Fig. 5:**
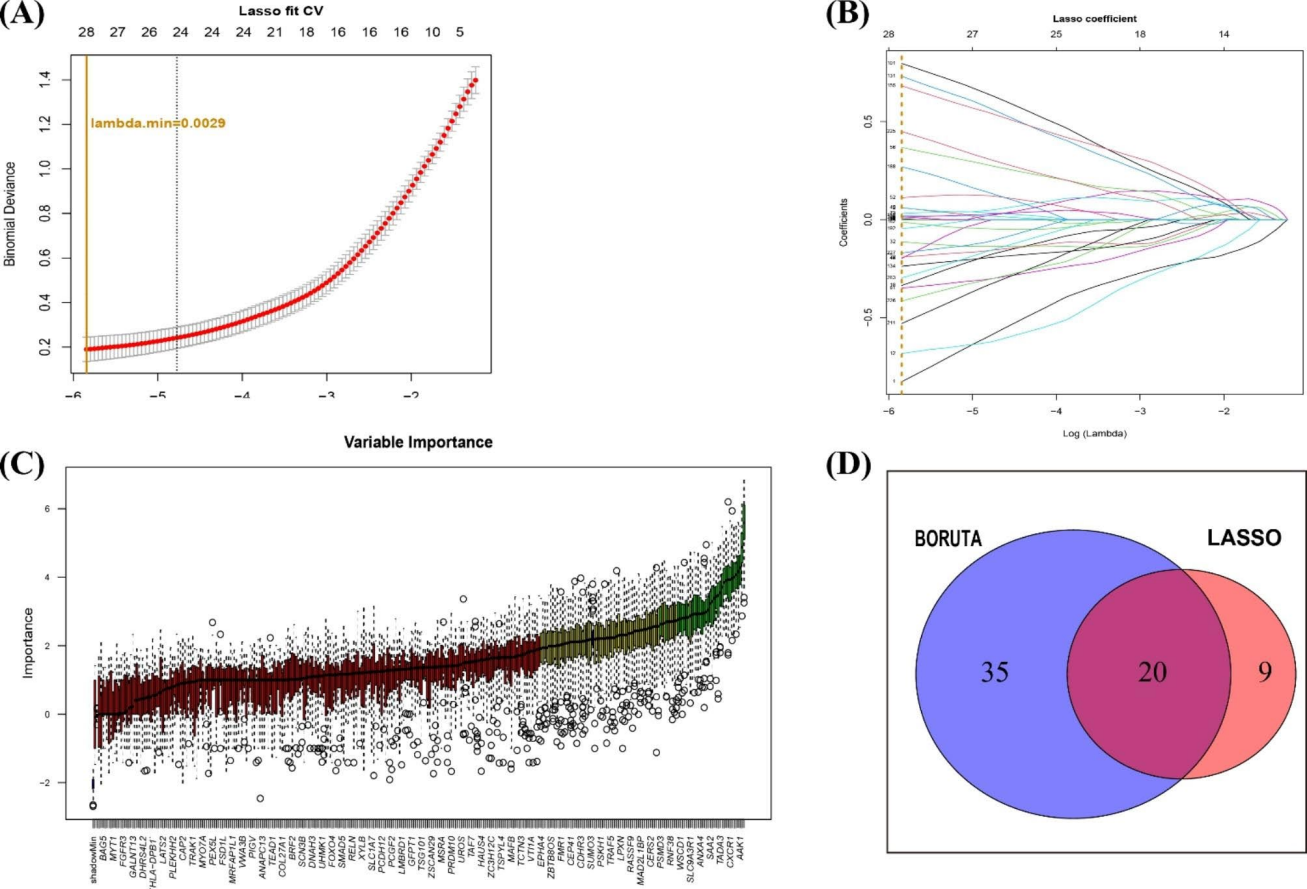
Screening of PCOS biomarkers. (**A**, **B**) LASSO regression analysis was used to screen for biomarkers. Different coloured lines represent different genes. (**C**) BORUTA algorithm for feature variable screening. (**D**) The intersection results of the two algorithms were obtained using a Venn diagram to yield candidate biomarkers

### Evaluation of the distributions of immune cell subtypes

ssGSEA revealed differences in the compositions of 28 immune cell subsets in the follicular microenvironment between healthy controls and patients with PCOS (Fig. 2A and Supplementary Table S2). Spearman co-expression analysis of 28 types of immune cells (Fig. 2B and Supplementary Table S3) showed that macrophages were significantly positively correlated with activated CD8^+^ T cells. Myeloid inhibitory cells were positively correlated with Tregs, Th1 cells, and activated DCs. Central memory CD4^+^ T cells were negatively correlated with Th2 cells. Additionally, compared with normal controls, patients with PCOS showed increased infiltration of central memory CD4^+^ T cells, central memory CD8^+^ T cells, and γδT cells, but decreased infiltration of effector memory CD4^+^ T cells and Th17 cells (Fig. 2C).

**Fig. 6:**
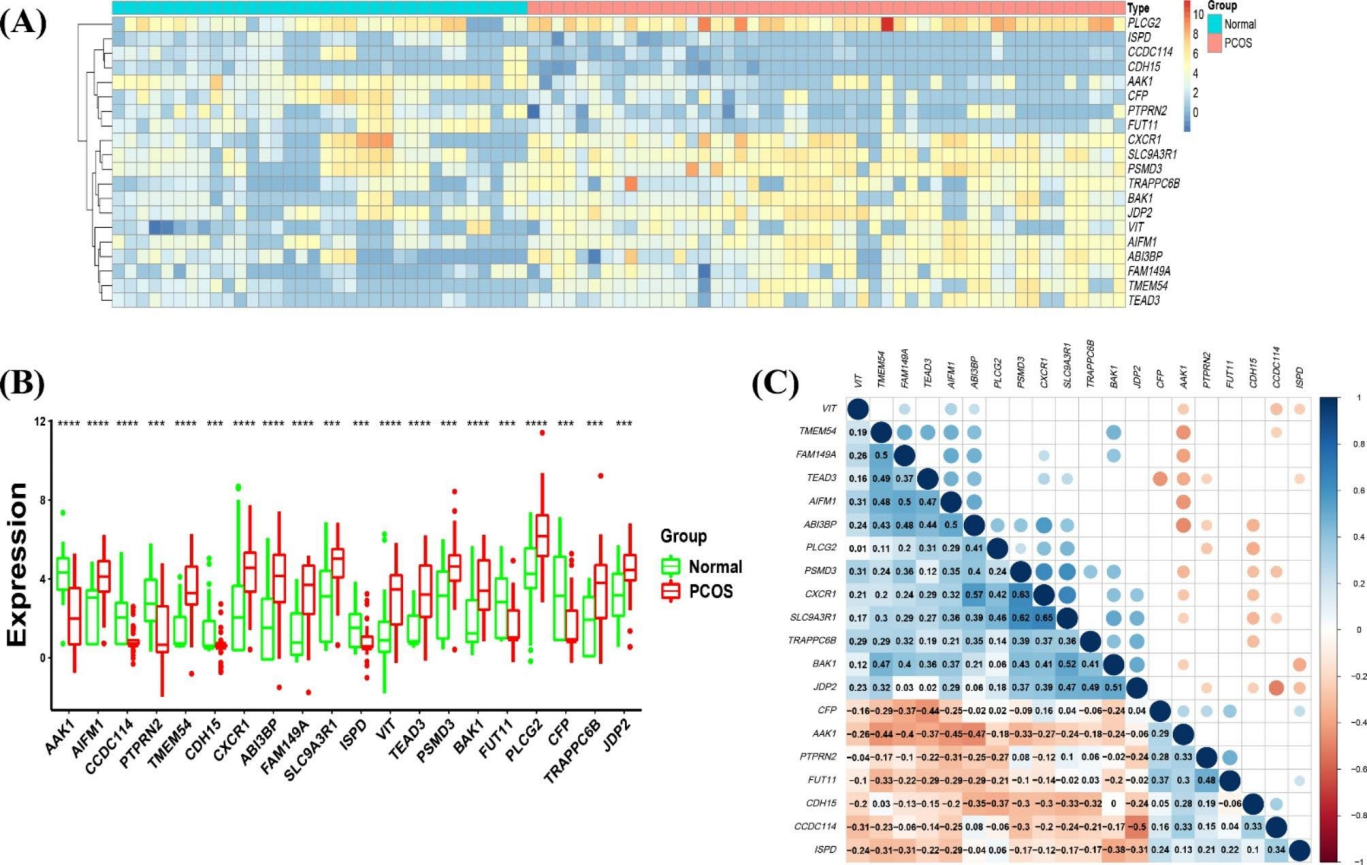
Expression of candidate biomarkers. (**A**) Heatmap of differences in the expression of candidate biomarkers between PCOS and normal control samples. The colours of the blocks represent the normalised gene expression levels in the samples. (**B**) Boxplot of differences in the expression of candidate biomarkers between groups. *** *p* < 0.001, and **** *p* < 0.0001. (**C**) Heatmap of the correlations between 20 candidate biomarkers. Colour blocks with correlation coefficients greater than 0.05 in the upper part of the figure are not displayed. Blue represents positive correlations, orange represents negative correlations, and darker colours indicate stronger correlations

**Fig. 7:**
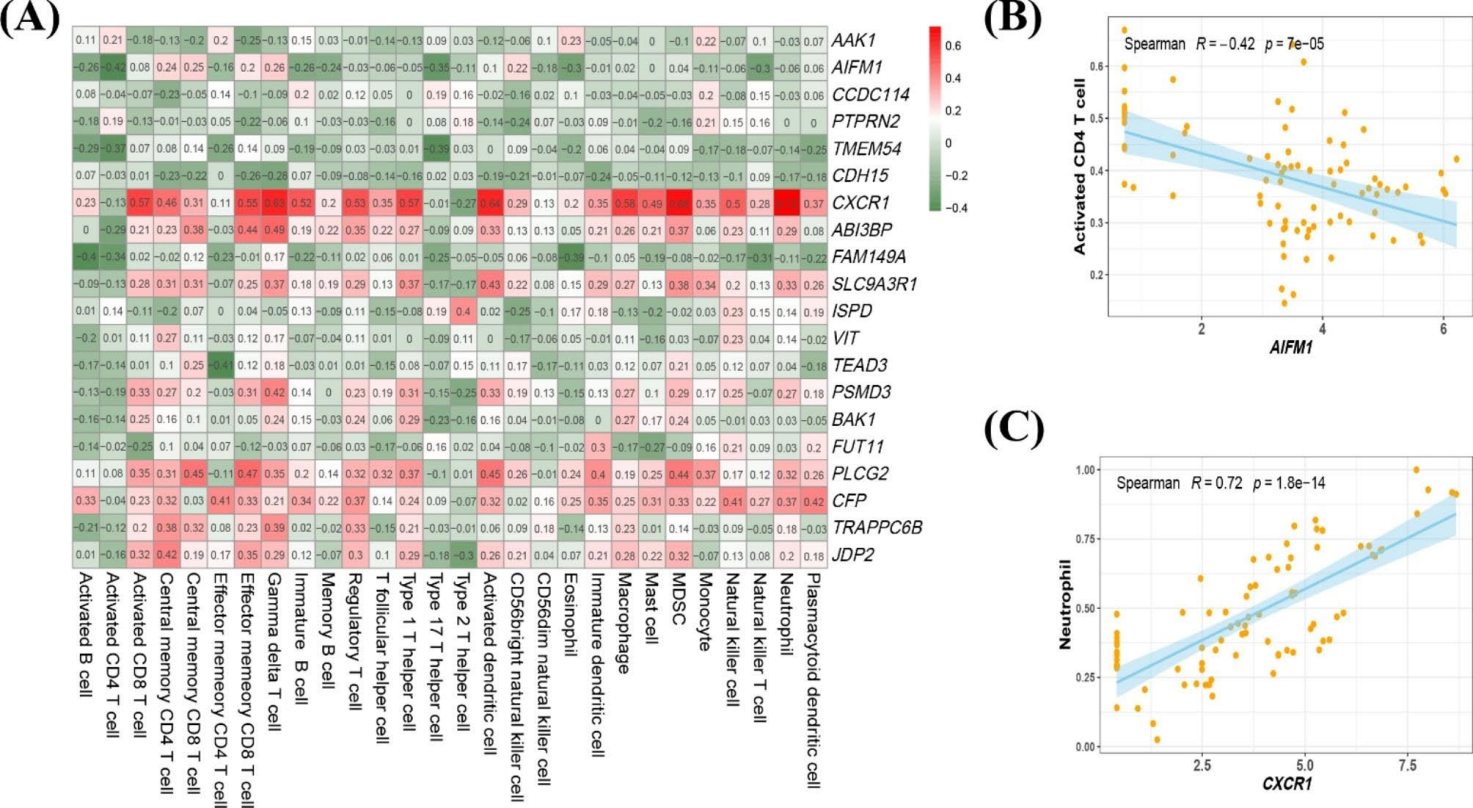
Correlations between candidate biomarkers and 28 types of immune cells. (**A**) Heatmap of the correlations between 28 immune cells and 20 candidate biomarkers. The colours of the blocks indicate the magnitude of the correlation. (**B**, **C**) Scatter plots of the two groups of correlations with the largest absolute values of positive and negative correlations

### Screening of DEGs

The above 83 samples were used to identify DEGs. In total, 325 DEGs were obtained, including 148 upregulated genes and 177 downregulated genes (Supplementary Table S4, Fig. 3A and B).

**Fig. 8:**
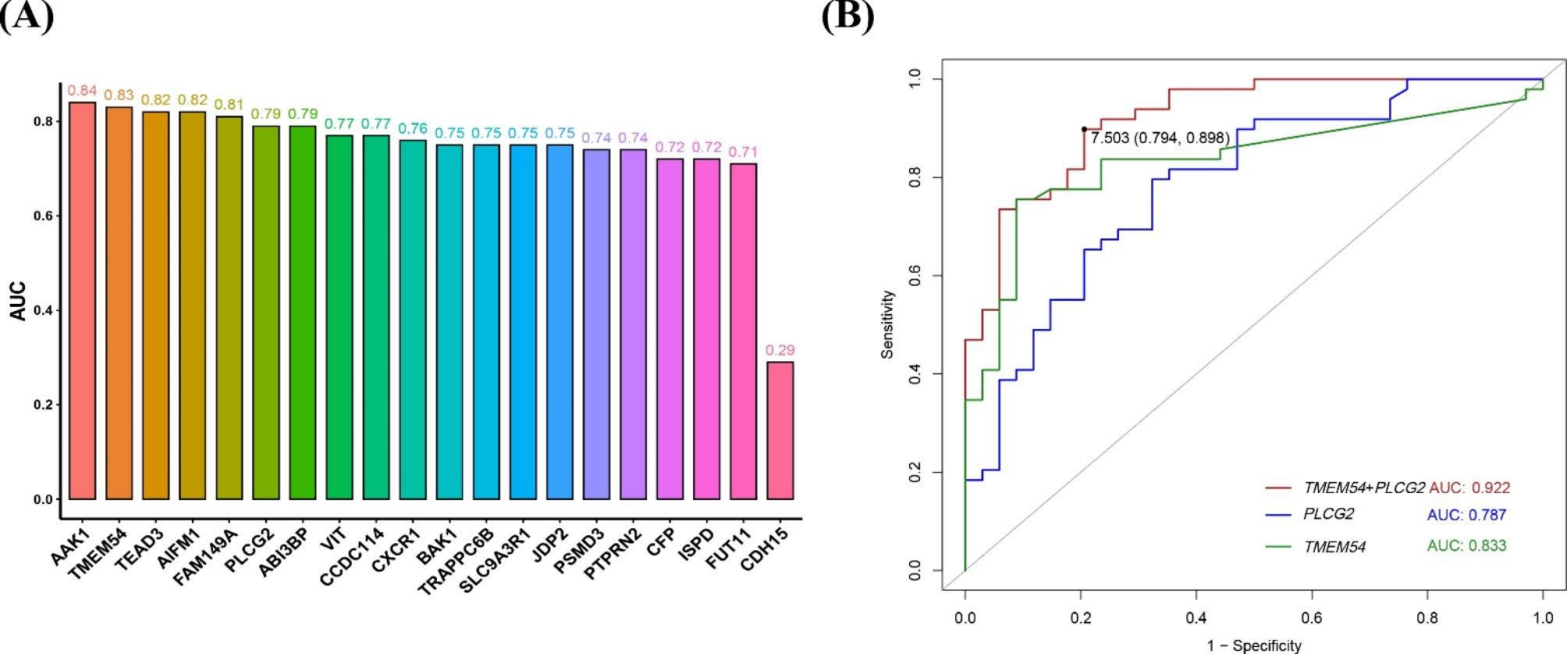
Predictive value of biomarkers. (**A**) The predictive value of 20 candidate genes for PCOS was evaluated using a bar graph of the AUC. An AUC value greater than 0.85 indicated that the model differentiating effect was satisfactory. (**B**) Diagnostic efficacy comparison of the ROC curves of *TMEM54* and *PLCG2* combined and separate

**Fig. 9:**
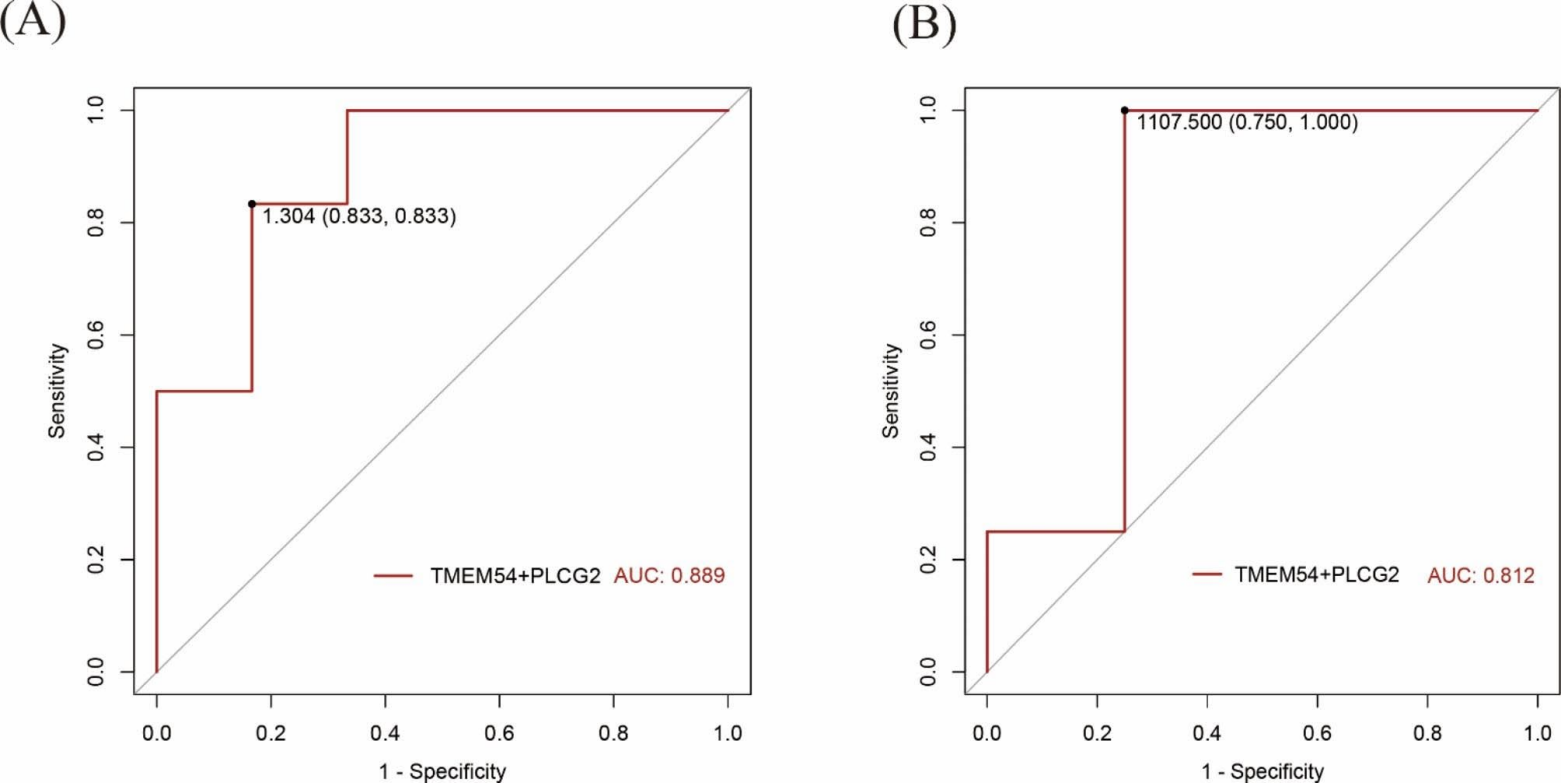
(**A**) Diagnostic efficacy of the ROC curves of *TMEM54* and *PLCG2* combined in RT-qPCR. (**B**) Diagnostic efficacy of the ROC curves of *TMEM54* and *PLCG2* combined in GSE193812.

### Functional enrichment analysis

DEG functional enrichment analysis was performed using GO, KEGG, and DO analysis. GO analysis showed that DEGs were mainly related to signal transduction and biological functions of cell activities, such as hormone receptor binding and ATP-dependent microtubule motility (Fig. 4A and Supplementary Table S5). KEGG analysis confirmed that DEGs were mainly related to growth metabolic signalling pathways, such as the Hippo signalling pathway, vesicle transportation, and vitamin digestion and absorption (Fig. 4B and Supplementary Table S6). DO analysis showed that DEGs were mainly related to autosomal recessive diseases and were also related to neurological and systemic haematological diseases (Fig. 4C and Supplementary Table S7).

### Screening of biomarkers

PPI network results for DEGs were obtained using STRING (Supplementary Figure S2). LASSO regression analysis was used to screen out 29 candidate biomarkers (Fig. 5A and B and Supplementary Table S8), the BORUTA algorithm was used to extract genes with high importance, and 55 candidate biomarkers were obtained (Fig. 5C and Supplementary Table S6). After analysis of the overlap of biomarkers obtained using the two algorithms, 20 candidate biomarkers were finally obtained (Fig. 5D and Supplementary Table S8), as follows: *AAK1*, *AIFM1*, *CCDC114*, *PTPRN2*, *TMEM54*, *CDH15*, *CXCR1*, *ABI3BP*, *FAM149A*, *SLC9A3R1*, *ISPD*, *VIT*, *TEAD3*, *BAK1*, *PSMD3*, *FUT11*, *PLCG2*, *CFP*, *TRAPPC6B*, and *JDP2*. Most genes were upregulated in PCOS, except *AAK1*, *CCDC114*, *PTPRN2*, *CDH15*, *ISPD*, *FUT11*, and *CFP* (Fig. 6A and B). Correlation analysis between genes showed that the *CXCR1* gene was significantly positively correlated with *SLC9A3R1* and *PSMD3*, whereas *CCDC114* was significantly negatively correlated with *JDP2* (Fig. 6C and Supplementary Table S9). Moreover, correlation analysis between the candidate biomarkers and 28 types of immune cells showed that *AIFM1* was negatively correlated with activated CD4^+^ T cells (R = − 0.42), and *CXCR1* was positively correlated with neutrophils (R = 0.72; Fig. 7 and Supplementary Table S9).

### Analysis of the predictive value of biomarkers

ROC analysis of candidate biomarkers showed that the combination of *TMEM54* and *PLCG2* had a high value for distinguishing PCOS from healthy women of reproductive age (AUC = 0.922). This indicated that the expression of *TMEM54* and *PLCG2* was related to PCOS and that combination of these two genes could be used as a biomarker for distinguishing PCOS from healthy women of reproductive age and for evaluating the efficacy of PCOS treatment (Fig. 8).

The predictive value of the selected biomarkers was tested using the external dataset GSE193812 and collected peripheral blood, and it was found that the biomarkers selected in this study still have good predictive ability both in the external dataset (AUC = 0.812) and in the peripheral blood samples (AUC = 0.889), as shown in Fig. 9.

## Discussion

PCOS is one of the most common ovo-derived endocrine diseases in women of reproductive age. Its basic pathophysiological features include hyperandrogenaemia and IR caused by disruption of the local internal environment, cytokine expression, and ovary function.

In this study, we identified DEGs between PCOS and normal controls and showed that the combination of *TMEM54* and *PLCG2* was a biomarker of PCOS. We also found that central memory CD4^+^ T cells, central memory CD8^+^ T cells, effector memory CD4^+^ T cells, γδT cells, and Th17 cells may influence the occurrence of PCOS. *PLCG2* was highly correlated with γδT cells and central memory CD4^+^ T cells.

The follicular microenvironment is composed of FF and granulosa cells (GCs). GCs regulate the local microenvironment of FF through various mechanisms, participate in the metabolism of oocytes, and protect oocytes from the invasion of components in the external environment [20]. The composition of the oocyte GC regulatory loop and follicular microenvironment is critical for the coordination of reproductive activities, and any changes in the composition of FF/GC molecules may affect the quality of oocytes [21]. Therefore, analysis of the immune infiltrating microenvironment of GCs can provide insights into the microenvironment of FF.

As the most important adaptive immune response cells in the immune system, T lymphocytes secrete various inflammatory and immunomodulatory molecules and are involved in regulating multiple ovarian functions, such as follicle formation, ovulation, and luteal degeneration. Activated lymphocytes secrete inflammatory cytokines, including IL-1, IL-6, IL-12, TNF-α, and insulin-like growth factor-1 [22]. In recent years, many domestic and foreign studies have reported that the levels of inflammatory factors, such as IL-6, TNF-α, and C-reactive protein, are higher in patients with PCOS than in normal women [23]. Moreover, inflammatory factors can mediate IR directly or indirectly through adipose tissue, suggesting that the occurrence and development of PCOS are closely related to inflammatory factors [24, 25]. Mature T cells can be categorised as CD4^+^ T cells or CD8^+^ T cells according to their CD molecular phenotype. Studies have shown that the ratio of CD4+/CD8 + is related to IL-2 [26, 27], IL-7 [28] and IL-16 [29]. CD4^+^ T cells and CD8^+^ T cells have different functions, which play synergistic or restrictive roles, and the ratio of CD4^+^/CD8^+^ T cells reflects changes in immune regulation.

Central memory T cells have the ability to expand, differentiate, and self-renew like stem cells and can differentiate unidirectionally into effector memory T cells and short-term effector T cells to prevent chronic infection and cancer [30]. In our study, central memory CD8^+^ T cells and central memory CD4^+^ T cells were increased in patients with PCOS, whereas effector memory CD4^+^ T cells were decreased, albeit without statistical significance. In the local ovarian microenvironment of patients with PCOS, abnormal CD4^+^/CD8^+^ T cell ratios may disrupt immune regulation. However, the differentiation of central memory T cells into effector memory T cells may also be blocked, resulting in increased central memory T cells and decreased effector memory T cells.

The functions of γδT cells were first discovered and studied in the field of autoimmune rheumatism. Although γδT cells are a highly conserved T-cell subpopulation, they have important implications in various aspects of immunobiology [31]. Animal models have shown that γδT cells regulate classical autoantigen reactive αβT cells and B cells [32, 33] and play independent pro-inflammatory roles via direct secretion of IL-17 [34], TNF-α, and interferon (IFN)-γ [35] in a non-antigen-driven pattern. Many studies have confirmed that the levels of TNF-α, IFN-γ, and IL-18 are significantly increased in patients with PCOS and are positively correlated with IR [24, 36, 37]. In this study, the numbers of γδT cells were higher in patients with PCOS than in normal controls, this observation may be related to the observed increases in TNF-α and IFN-γ secretion by high numbers of γδT cells. Notably, CD3^+^CD4^−^CD8^−^ γδT cells are increased in women with recurrent abortion, contributing to foetal loss by regulating transforming growth factor-β and IL-17 secretion and promoting inflammation [38]. The risk of abortion after pregnancy in patients with PCOS is more than three times higher than that in normal women, which may be related to the presence of a chronic inflammatory state and autoimmune disorders, such as active autoimmunity.

The high correlation between the CXC motif chemokine receptor 1 (*CXCR1*) gene and various immune cells was notable, and the correlation between *CXCR1* and neutrophils reached R = 0.72. The protein encoded by the *CXCR1* gene is a member of the G-protein-coupled receptor family and acts as a receptor for IL-8. IL-8 is a powerful neutrophil chemokine that binds to receptors and promotes neutrophil activation. *CXCR1* binds to IL-8 with high affinity and transduces signals through a second messenger system activated by G proteins [39, 40]. This explains the results of our analysis from a mechanistic level and supports the accuracy of our results.

One of the selected biomarkers, transmembrane protein 54 (*TMEM54*), is a member of the transmembrane protein family, which contains many proteins with unknown functions. Studies have shown that *TMEM* expression is different in tumour tissues than in adjacent healthy tissues, and some *TMEM* family members have been identified as potential prognostic biomarkers in different types of tumours [41]. In addition, *TMEM* proteins are tumour suppressors or oncogenes and have been shown to be associated with tumour progression and invasion [42, 43] or chemotherapy resistance [44, 45]. Although there are few studies on *TMEM54*, our current findings suggest that the *TMEM54* gene may have important roles in the development of PCOS; further studies are warranted.

Phospholipase C gamma 2 (*PLCG2*) is a transmembrane signalling enzyme that is an important driver of many immunological aetiological diseases, such as inflammation, autoimmune diseases, immunodeficiencies, and allergies, as well as haematological malignancies. Some studies have demonstrated that point mutations in the *PLCG2* gene may be an important cause of severe spontaneous inflammation and autoimmunity [46]. In a meta-analysis of gene expression in patients with rheumatoid arthritis (RA), *PLCG2* was found to be upregulated in several datasets, including many pathways associated with RA inflammatory responses, e.g., inflammasome activation, platelet aggregation, and activation, indicating that *PLCG2* is a potential target for the control of RA inflammation [47]. Moreover, *PLCG2* is important in bone marrow cells, including monocytes, macrophages, NK cells, DCs, and mast cells, possibly because it promotes downstream signalling involving Fc receptors [48]. In a bioinformatics analysis of the tumour microenvironment in soft tissue sarcoma, *PLCG2* was found to be an indicator of the tumour microenvironment and patient prognosis [49]. Additionally, CD8^+^ T cells, γδT cells, monocytes, and M1 macrophages were shown to be positively correlated with *PLCG2* expression, consistent with our current results.

There were some limitations to this study. First, this study was only carried out from the perspective of gene transcriptome, and multi-omics and mechanistic studies were not performed. Further validation of our bioinformatics results through *in vitro* and *in vivo* experiments and clinical practice is needed.

In summary, in this study, ssGSEA was used for the first time to analyse immune infiltration into the follicle microenvironment in patients with PCOS. Central memory CD4^+^ T cells, central memory CD8^+^ T cells, effector memory CD4^+^ T cells, γδT cells, and Th17 cells may be involved in the occurrence of PCOS. In addition, differences in gene expression in ovarian tissues between patients with PCOS and healthy women of reproductive age were determined, and our findings showed that the combination of TMEM54 and PLCG2 was a biomarker of PCOS. PLCG2 was shown to be highly correlated with γδT cells and central memory CD4^+^ T cells. These findings provide a basis for further research on the immunological pathogenesis of PCOS.

## Electronic supplementary material

Below is the link to the electronic supplementary material.

**Additional file 1:** Supplementary Tables

**Additional file 2:** Supplementary Figures

## Data Availability

The datasets used and/or analysed during the current study are available from NCBI Gene Expression Omnibus (GEO). GSE84958 https://www.ncbi.nlm.nih.gov/geo/query/acc.cgi?acc=GSE84958. GSE106724 https://www.ncbi.nlm.nih.gov/geo/query/acc.cgi?acc=GSE106724. GSE114419https://www.ncbi.nlm.nih.gov/geo/query/acc.cgi?acc=GSE114419. GSE137684 https://www.ncbi.nlm.nih.gov/geo/query/acc.cgi?acc=GSE137684. GSE193812 https://www.ncbi.nlm.nih.gov/geo/query/acc.cgi?acc=GSE193812.
