# Supplementary figures and images for: Identification of immune cell infiltration and effective biomarkers of polycystic ovary syndrome by bioinformatics analysis
Source: BMC Pregnancy Childbirth. 2023 May 24;23:377. doi: 10.1186/s12884-023-05693-4 (PMC10207797; doi:10.1186/s12884-023-05693-4)

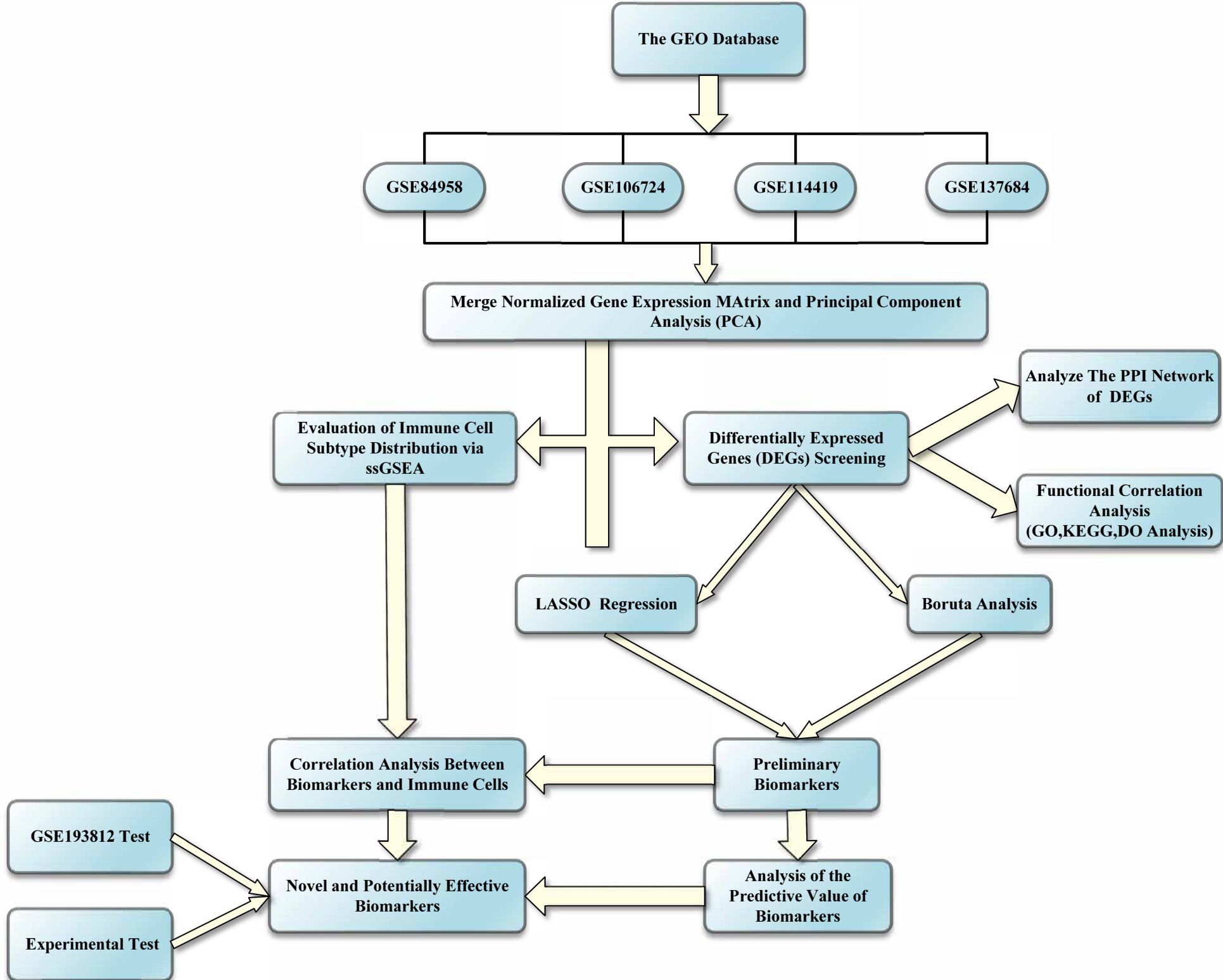

**Supplementary Figure F1 The Flow Chart**

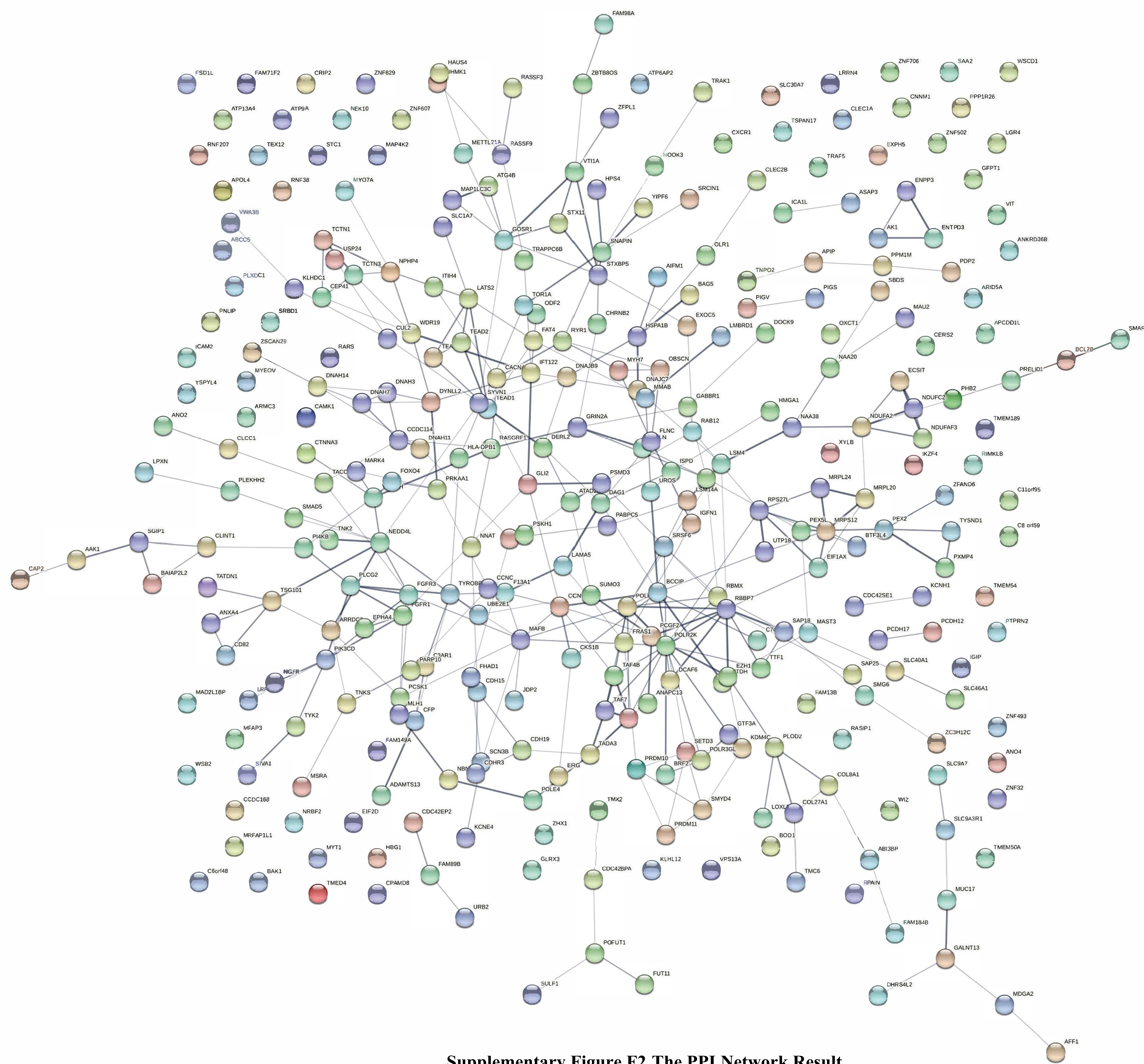

Supplement: Supplementary file 2 — Additional file 2: Supplementary Figures [file 12884_2023_5693_MOESM2_ESM.pdf]
